# New Developments at Leicester

**Published:** 1914-08-08

**Authors:** 


### New Developments at Leicester.

AT the recent half-yearly meeting of the Governors of the Leicester Royal Infirmary, presided over by Sir Arthur Hazlerigg, Bart., regret was expressed at the absence of the chairman, Sir Edward Wood, and a message was sent conveying the governors' hope for his restoration to health.

The income was £10,386, compared with £9,078 last year, and the total expenditure stood at £14,135, against £12,626. Satisfaction was expressed with the large income received as the result of parades, fêtes, and demonstrations. The children's hospital is now a feature of the institution's work, and it is hoped that the £3,700 required to complete the cost of reconstruction will be forthcoming.

In order to keep pace with the rapidly growing demands for a pathological department, the honorary medical staff had strongly urged the board to build and equip a new department adjacent to the mortuary (which latter building it was proposed to reconstruct and refit), at an approximate cost of £4,000, and it was proposed to commence the work without delay.

The board's report showed 2,095 in-patients admitted during the six months, 21,776 out-patients, as compared with 22,085, 21,659 casualty cases, against 22,208, and 2,394 operations, against 2,453. Statistics disclosed the fact that 50 per cent. of the total in-patients of sixteen years and upwards admitted to the infirmary during the past year were insured persons under the National Insurance Act, the cost of whose treatment, and of the large number of insured out-patients and accident cases, was not less than £8,000. The figures carried with them the conviction that the Insurance Committee and the Approved Societies would give the matter close consideration with a view to treatment of urgent conditions being secured to insured persons on definite lines, and not left to casual methods.

A PRIVATE NURSING HOME.

The board have taken over premises formerly used as a private nursing home on the Aylestone Road, and they propose at a later date to undertake private nursing in Leicester, their object being not to work on competitive lines with other institutions, but to provide for the certificated nurses of the institution a course of private nursing, and thus enhance the value of the institution as a training school for nurses.

